# Different In Vitro Models of Chronic Myeloid Leukemia Show Different Characteristics: Biological Replicates Are Not Biologically Equivalent

**DOI:** 10.1002/cbin.70007

**Published:** 2025-03-01

**Authors:** Alessia Cavalleri, Besjana Xhahysa, Silvia Mutti, Rosalba Monica Ferraro, Elena Laura Mazzoldi, Mirko Farina, Alessandro Leoni, Luca Garuffo, Federica Trenta, Federica Re, Vera Radici, Eugenia Accorsi Buttini, Enrico Morello, Gabriele Magliano, Valeria Cancelli, Silvia Clara Giliani, Michele Malagola, Domenico Russo, Simona Bernardi

**Affiliations:** ^1^ Department of Clinical and Experimental Sciences University of Brescia, Unit of Blood Diseases and Bone Marrow Transplant, ASST Spedali Civili Brescia Italy; ^2^ Centro di Ricerca Emato‐Oncologica AIL (CREA), ASST Spedali Civili Brescia Italy; ^3^ Department of Molecular and Translational Medicine University of Brescia, “Angelo Nocivelli” Institute for molecular medicine, ASST Spedali Civili Brescia Italy; ^4^ Hematology, ASST Spedali Civili Brescia Italy; ^5^ National Center for Gene Therapy and Drug based on RNA Technology—CN3 Padova Italy

**Keywords:** chronic myeloid leukemia (CML), digital PCR, K562, KCL22, LAMA84, STAMP inhibitor, tyrosine kinase inhibitors (TKIs)

## Abstract

Chronic Myeloid Leukemia (CML) is characterized by the BCR::ABL1 fusion gene, driving uncontrolled myeloid cell proliferation. Furthermore, metabolic dysregulation contributes to disease progression. Despite the efficacy of tyrosine kinase inhibitors (TKIs), unresolved clinical needs persist, necessitating refined preclinical models. This study compared responses of three commonly used CML cell lines (K562, LAMA84, KCL22) to five TKIs (imatinib, nilotinib, dasatinib, bosutinib, ponatinib) and a Specifically Targeting the ABL Myristoyl Pocket (STAMP) inhibitor commonly used in clinical settings. Using morphological assessments, viability and metabolic activity assays, glutamate intake evaluations, and gene expression analyses we observed distinct responses among cell lines. TKIs and STAMP inhibitor treatments showed varying impacts on morphological features, cell viability, metabolic activity, and gene expression profiles, highlighting significant differences in cellular responses. This emphasizes the necessity of considering cellular heterogeneity in CML research. This comprehensive comparison provides valuable insights for refining preclinical models and enhancing translational relevance in CML research and treatment development. Understanding the diverse responses of CML cell lines to TKIs and STAMP inhibitor facilitates the selection of appropriate models for specific research questions, ultimately improving the accuracy and reliability of preclinical studies in CML.

**Abbreviations**
ABL1: Abelson murine leukemia viral oncogene homolog 1
BC: blast crisis
BCR: breakpoint cluster region
CML: chronic myeloid leukemia
CP: chronic Phase
dPCR: digital Polymerase Chain Reaction
FDA: Food and Drug Administration
GAPDH: glyceraldehyde‐3‐phosphate dehydrogenase
PB: peripheral blood
PCR: polymerase chain reaction
Ph: Philadelphia chromosome
STAMP: specifically targeting the ABL myristoyl pocket
STR: short tandem repeat
TKIs: tyrosine kinase inhibitors

## 1 Introduction

Chronic Myeloid Leukemia (CML) is a myeloproliferative disorder characterized by the uncontrolled proliferation and accumulation of several types of myeloid precursor cells both in the peripheral blood (PB) and bone marrow (BM). The genetic cause of the pathology is a reciprocal translocation between chromosomes 9 and 22 (t9;22) (q34; q11) that results in the fusion of the breakpoint region (BCR) on chromosome 22 with the Abelson murine leukemia viral oncogene homolog 1 (ABL1) tyrosine kinase on chromosome 9, generating the Ph+ chromosome and the *BCR::ABL1* fusion gene (Nowell and Hungerford 1961). The consequent BCR::ABL1 protein, that is present in different isoforms, is a constitutively active tyrosine kinase capable of affecting several important signaling pathways related to cell adhesion, survival, proliferation, and differentiation (Bernardi et al. 2019; Clarke and Holyoake 2017).

In this context, metabolic alterations are pivotal for disease progression. Dysregulated glucose metabolism, marked by increased glucose uptake and glycolytic activity, meets leukemic cells' high energy demands. Also, the glutamine pathway, already identified as altered and a potential therapeutic target in Acute Myeloid Leukemia (Gregory et al. 2019), is similarly disrupted at the stem/progenitor cell level in CML (Poteti et al. 2021). Additionally, abnormalities in lipid and amino acid metabolism contribute to leukemogenesis. Notably, disruptions in glutamate metabolism are significant, impacting essential cellular processes linked to CML pathogenesis, such as cell proliferation and therapy resistance (Jiye et al. 2010; Li et al. 2022).

The natural course of untreated CML, according to the latest 2022 WHO guidelines, can be categorized into two phases: the Chronic Phase (CP) and the Blastic Crisis (BC). The CP is typically where diagnosis occurs, while BC usually occurs 3–5 years after diagnosis (Chereda and Melo 2015; Khoury et al. 2022).

In the late 1990s, recognizing CML's dependency on BCR::ABL expression, several ABL tyrosine kinase inhibitors (TKIs) were developed and currently represent the first line of treatment for CML. The first TKI approved by Food and Drug Administration (FDA) for use in CML patients was imatinib in 2001, which inhibits constitutive tyrosine kinase activity and returns BCR::ABL1 to its inactive conformation by binding tightly to its ATP binding site. Subsequently, second‐generation inhibitors, such as nilotinib, dasatinib, and bosutinib, and a third‐generation inhibitor specific for T315I mutation, ponatinib, were generated. Contrary to first‐generation inhibitors, second‐generation inhibitors can bind to both the active and inactive forms of BCR::ABL1 and are approximately 1000 times more effective than imatinib against resistant BCR::ABL1 mutations (Amir and Javed 2021; Russo et al. 2023). The advent of asciminib, a first‐in‐class STAMP (Specifically Targeting the ABL Myristoyl Pocket) inhibitor, has further expanded the therapeutic landscape of CML, providing a novel mechanism of action to overcome resistance and improve patient outcomes (Manley et al. 2020).

Despite the effectiveness of available therapies, there are still unresolved clinical needs in the specific setting of CML (Bernardi et al. 2017, 2018; Kockerols et al. 2022, 2024). Thus, having preclinical models that accurately predict clinic performance is crucial to effectively translating laboratory results to patients (Zizioli et al. 2019, 2021). In this context, in the last few years, an increasingly extensive panel of human CML Ph+ cell lines have been generated and described (Table 1) (Drexler et al. 1999).

**Table 1:** CML‐derived cell lines. In particular, name of cell line, cell type, sex and age of patient at time of establishment of cell line, disease status, year of establishment of cell line, source of specimen as indicated in the original publication and type of BCR,ABL1 fusion are indicated (BC = blastic crisis, BCP = B‐Cell Precursor, B‐LCL = B‐lymphoblastoid cell line (EBV + ), BM = bone marrow, BMT = bone marrow transplantation, F = female, M = male, NA = not available, R = relapse, PB = peripheral blood, PE = pleural effusion).

Cell line	Cell type	Age/sex of patient	Disease status	Year establishment	Specimen site	*BCR::ABL1* transcript fusion type
AP‐217	Ery‐megakaryocytic	42 M	BC	1992	PB	e14a2
AR230	Myelocytic	52 F	BC	1991	PB	e19a2
BV173	BCP	45 M	BC	1980	PB	e13a2
CML‐C‐1	Myelocytic	42 F	BC	1990	PB	
CML‐T1	T‐cell	36 F	BC	NA	PB	e13a2
EM‐2/EM	Myelocytic	5 F	2nd R (BMT)	1982	BM	e14a2
GM/SO	Myelocytic	50 F	BC	1988	BM	e14a2
IMS‐BC1	Myelocytic	46 F	BC	1991	BM	e14a2
IMS‐BC2	Myelocytic	50 M	BC	1995	PB	e13a2/e14a2
IMS‐BC3	Myelocytic	35 F	BC	1995	PB	e13a2/e14a2
JK‐1	Erythrocytic	62 M	BC	1987	Tumor	e13a2
JURL‐MK1	Ery‐megakaryocytic	73 M	BC	1993	PB	e14a2
JURL‐MK2	Ery‐megakaryocytic	73 M	BC	1993	PB	e14a2
K562	Erythrocytic	53 F	BC	1970	PE	e14a2
KASUMI‐4	Myelocytic	6 F	BC	1993	PB	e13a2
KBM‐5	Monocytic	67 F	2nd BC	NA	PB	e14a2
KBM‐7	Myelocytic	39 M	BC	1984	BM	e13a2
KCL22	Myelocytic	32 F	BC	1981	PE	e13a2
KH88‐B4D6	Erythrocytic	70 M	BC	1988	PB	e14a2
KH88‐C2F8	Erythrocytic	70 M	BC	1988	PB	e14a2
KOPM‐28	Myelocytic	64 F	BC	1982	PB	e14a2
KT‐1	Myelocytic	32 M	BC	1991	PB	e13a2
KU‐182	Myelocytic	38 M	BC	1981	PB	e14a2
KYO‐1	Myelocytic	22 M	BC	1981	PB	e13a2
LAMA84/87/88	Ery‐megakaryocytic	29 F	BC	1984	PB	e14a2
MC3	Megakaryocytic	51 F	BC	1986	PB	e14a2
MEG‐01	Megakaryocytic	55 M	BC	1983	BM	e13a2
MEG‐A2	Megakaryocytic	24 M	BC	1991	PB	e14a2
MOLM‐1	Megakaryocytic	41 M	BC	1988	BM	e13a2
NALM‐1	BCP	3 F	BC	1975	PB	e14a2
NS‐Meg	Megakaryocytic	44 F	BC	1992	PB	e14a2
PhB1	B‐LCL	67 M	BC	1991	BM	e14a2
RM10	Erythrocytic	44 F	BC	1990	BM	M‐bcr
RWLEU‐4	Monocytic	NA	BC	NA	PB	e13a2
SAM‐1	Ery‐megakaryocytic	22 M	BC	1987	PB	e14a2
T‐33	Megakaryocytic	62 F	BC	1985	PB	e14a2
TK‐6	T‐cell	30 M	R (BMT)	1992	PE	e14a2
TS9;22	Megakaryocytic	56 M	BC	1992	PB	e14a2
Y‐1K	Ery‐megakaryocytic	57 F	BC	1990	PB	e14a2
YN‐1	Erythrocytic	35 M	BC	1980	PB	e14a2
YOS‐M	Myelocytic	77 M	BC	1990	PB	e13a2
YOS‐B	B‐LMC	77 M	BC	1990	PB	NA
YS9;22	Megakaryocytic	23 F	BC	1992	PB	e14a2

Primarily used as an initial high‐throughput tool to validate potential therapeutic targets and screen drug candidates, immortalized cell lines provide a continuous source of reproducible cellular material and are amenable to an extensive variety of in vitro assays. Moreover, the versatility of various in vitro Ph+ models, due to the different characteristics of the patients from whom they are derived, has offered the possibility of analyzing the biology underlying leukemogenesis and the role of BCR::ABL1, and still allows different responses to treatments to be assessed (Clarke and Holyoake 2017).

Three of the most used cell lines for CML research are K562, LAMA84, and KCL22. Specifically, K562 cells were derived from the pleural effusion of a 53‐year‐old woman during BC, LAMA84 cells originated from the peripheral blood of a 29‐year‐old woman who had previously undergone 5 years of busulfan treatment, and KCL22 cells were isolated from the pleural effusion of a 32‐year‐old woman experiencing BC (https://www.Dsmz.de/Collection/Catalogue/Human-and-Animal-Cell-Lines/Catalogue). Additional information on the cell lines can be found in Supporting Information.

All three lines mentioned have been extensively used in research. K562 cells were mainly employed in studies on immune system disorders, immunology, and in vitro assays involving Natural Killer cells. They have also been utilized in research investigating the protective role of the Differentiation Cluster‐39 (CD39) against DNA damage and in exploring the relevance of a novel cell separation approach called “Slice and Dice Sorting” (Aho et al. 2016; Osborne et al. 2015). Similarly, KCL22 cells were employed in research on immune system disorders and in sensitivity studies to TKIs (Ohmine et al. 2003). In contrast, LAMA84 cells, were studied to probe glucose uptake and the non‐oxidative glycolytic metabolic phenotype in cells that are either resistant or not resistant to specific TKIs (Kominsky et al. 2009).

Despite these cell lines being derived from different biological sources and patients, they are often used interchangeably and considered biological replicates in experimental studies. However, to date, no studies have been conducted to define the specific characteristics of individual lines and how these may influence the results of various experiments. Only one study has examined the proteomic profiles of these CML cell lines treated with imatinib, revealing partial differences and identifying proteins consistently upregulated or downregulated across models, shedding light on their roles in CML pathogenesis (Fontana et al. 2007).

Therefore, this study aims to compare the responses of these three most used in vitro CML models (K562, LAMA84, and KCL22 cell lines) when treated with five commonly used TKIs (imatinib, nilotinib, dasatinib, bosutinib, and ponatinib) and a STAMP inhibitor (asciminib) using a multiparametric approach.

## 2 Materials and Methods

### 2.1 Culture Conditions and Treatments

For this study, three different CML in vitro models were used: K562 (DSMZ, German Collection of Microorganisms and Cell Cultures, Catalogue Number: ACC10), LAMA84 (DSMZ, German Collection of Microorganisms and Cell Cultures, Catalogue Number: ACC168), and KCL22 (DSMZ, German Collection of Microorganisms and Cell Cultures, Catalogue Number: ACC519) cell lines. Specifically, TKI‐sensitive cell lines were selected for our investigations (K562‐s, LAMA84‐s, and KCL22‐s, respectively). For Authenticity, short tandem repeat (STR) analysis were conducted by DSMZ according to the global standard ANSI/ATCC ASN‐0002.1‐2021 (2021) and resulted in an authentic STR profile of the reference STR database. Mycoplasma contamination was eliminated before shipping using Ciprobay (ciprofloxacin), and later excluded by negative DAPI staining, microbiological culture, and PCR assays.

Cells were grown in complete medium consisting of RPMI 1640 (ThermoFisher Scientific) supplemented with 0.01% penicillin/streptomycin (stock solution 10,000 IU/mL penicillin and 10 mg/mL streptomycin, Sigma Aldrich), 0.0005% fungizone (stock solution 250 mg/mL, Sigma Aldrich), and 10% FBS (Sigma Aldrich). Before experimentation, the presence of Mycoplasma in cells cultures was excluded by Polymerase Chain Reaction (PCR) assays.

Following expansion in T75 cm^2^ flasks, one 48‐well and two 96‐well plates for each cell line were prepared for specific applications. Cells were seeded at a density of 25,000 cells/well in 250 μL of complete medium for the 48‐well plate or in 100 μL for the 96‐well plates and then cultured for 48 h.

Five TKIs from the Adult Marrow Transplant Centre of the ASST Spedali Civili of Brescia were tested at their corresponding concentrations: imatinib (Gleevec, Novartis) 0.64 μM; nilotinib (Tasigna, Novartis) 5 μM; dasatinib (Sprycel1, Bristol‐Myers Squibb) 1.3 nM; bosutinib (Bosulif, Wyeth) 250 nM; ponatinib 4 nm (Iclusig, Ariad Pharmaceutics). The STAMP inhibitor asciminib (Scemblix, Novartis) was obtained from the Hematology Department of the ASST Spedali Civili of Brescia and was used at a concentration of 10 nm. Drug concentrations, that aimed to simulate the in vitro effects of the dose administered in clinical practice, were identified in literature, and adapted according to our experience (Baty 2022; Okabe et al. 2024b; Peixoto‐da‐Silva et al. 2018; Tusa et al. 2020; Willig et al. 2020; Zhang et al. 2022). Since all drugs stock solutions were realized in DMSO (Sigma‐Aldrich), this condition was also added at a concentration of 0.33% of the final volume and considered as control (Tusa et al. 2020).

**Figure 1:**
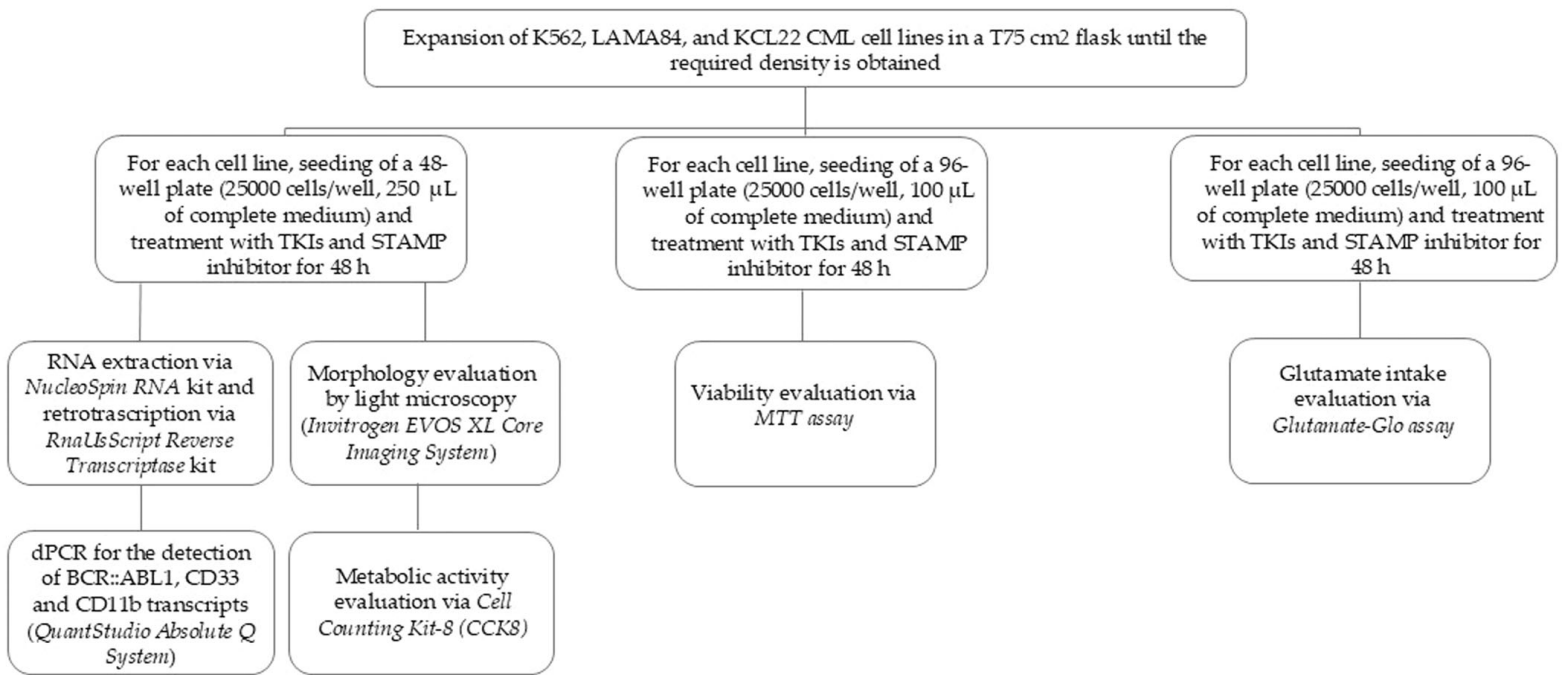
Flow chart representing the experimental design of the study.

Each condition was repeated in triplicate, for a total of 24 conditions for each cell line. The in vitro experimental investigations scheme is shown in the flow chart below (Figure 1).

### 2.2 Dimensional and Morphological Analyzes

To analyze morphological variations among the different treatment groups and the different cell lines investigated, images were acquired at the end of the culture (and thus, 48 h after seeding) through the Invitrogen EVOS XL Core Imaging System (ThermoFisher Scientific). Specifically, seven different panels were produced, relating to each treatment condition, in which the three different in vitro models were compared. In each panel, two images of each line were included at 4× zoom, for a larger view of the well, and 20× zoom, for visualization of specific details and cell morphology. In the case of 20× zoom, well areas were selected that best represented the cellular state, including color, shape, size, and the formation of dead cell clusters. Subsequently, to evaluate the dimensional and morphological changes between the various treatment strategies, 10 measurements were made per each panel, and using the ImageJ software (Version 1.54), the average area, diameter, and circularity resulting from each treatment in each line were assessed.

### 2.3 Viability and Metabolic Activity in Vitro Assays

Cell viability was assessed after 48 h of treatment using a commercial kit, the MTT assay (Sigma‐Aldrich). The manufacturer's instructions were followed for the execution. Briefly, 10 μL of MTT solution (obtained by adding PBS at pH 7.4 to the MTT reagent) was added to each treatment condition (whose volume was 100 μL).

After 4 h of incubation, 100 μL of MTT stop solution (made by adding isopropanol with 0.04 N HCl) was added to each condition and the absorbance was read at 570 nm using a Tecan Infinite 200 spectrophotometer (Tecan Group Ltd.).

Metabolic activity resulting from the different treatments was assessed after 48 h using another commercial enzyme‐based Cell Counting Kit‐8 (Sigma‐Aldrich). Again, the manufacturer's instructions were followed for the procedure. Briefly, a solution containing complete culture medium, and 10% CCK‐8 reagent was added to each condition. After a 2‐h incubation, absorbance was measured at 460 nm using a Tecan Infinite 200 spectrophotometer (Tecan Group Ltd.).

For both assays, absorbance was directly proportional to viability and metabolic activity, respectively. In particular, cell viability was calculated as percentage in respect to controls. Conversely, metabolic activity was expressed in terms of the number of metabolically active cells. For this purpose, a calibration curve was established for each cell line.

### 2.4 Glutamate Intake Evaluation Assay

Glutamate intake following each treatment was assessed using the in vitro bioluminescence Glutamate‐Glo Assay (Promega). For this purpose, the extracellular level of the metabolite in the culture medium, which inversely correlates with the intracellular concentration, was analyzed for each treatment. In summary, 5 µL from each condition was collected and added to 95 µL of PBS. Following this, 50 µL of every sample was combined with 50 µL of Glutamate Detection Reagent, which was prepared as follows for each reaction: 50 µL of Luciferin Detection Solution, 0.25 µL of Reductase, 0.25 µL of Reductase Substrate, 1 µL of Glutamate Dehydrogenase, and 1 µL of NAD. After shaking for 30–60 s and incubating for 1 h at room temperature, luminescence was measured by a Tecan Infinite 200 spectrophotometer (Tecan Group Ltd.).

### 2.5 Cellular RNA Extraction and cDNA Synthesis

RNA extraction was performed using NucleoSpin RNA extraction kit (Machery Nagel) following the manufacturers' instructions. The total RNA was eluted in a volume of 50 µL. To obtain cDNA, the extracted RNAs were immediately quantified using a Qubit RNA high‐sensitivity kit (ThermoFisher Scientific) and retrotranscribed via RNAUsScript Reverse Transcriptase Kit (LeGene Biosciences). Briefly, to obtain the highest cDNA concentration, the maximum possible volume of RNA (8 μL) was retrotranscribed. Then, 200 U of Reverse transcriptase (Superscript II), 10 mM of dNTP, 50 ng/μL of Random hexamer, and 5× RT buffer were added to a final volume of 20 μL. This step was followed by three subsequent incubations: at room temperature for 10′, at 42°C for 45′, and at 99°C for 30′, with a final holding period at 4°C. All procedures were executed by Applied Biosystems Veriti Thermal Cycler (ThermoFisher Scientific). The cDNA was quantified using Qubit ssDNA kit (ThermoFisher Scientific) and then stored at ‐20°C until further analysis.

### 2.6 dPCR Analysis for *BCR::ABL1*, *CD33* and *CD11b*

For the absolute quantification of *BCR::ABL1* (the CML hallmark), *CD33* (a monocyte marker) and *CD11b* (a neutrophil marker) transcripts, the QuantStudio Absolute Q dPCR system in multiplex (Applied Biosystems, Software 6.2.1) was applied. Specifically, the fluorochromes FAM, VIC, and ABY were specifically used to tag the *BCR::ABL1* transcript probe (Bernardi et al. 2022, 2024; Bernardi et al. 2019; Fava et al. 2021; Soverini et al. 2020; Zanaglio et al. 2020), *CD33* probe, and *CD11b* probe, respectively. This ensured that there was no overlap in signals across the three different wavelengths. The results for the transcripts investigated were subsequently normalized by quantifying the housekeeping gene *GAPDH*. This normalization method was designed to eliminate the pre‐analytical variable associated with extraction and retrotranscription among the triplicates of each condition.

Each sample was prepared as follows: 1.8 μL of Absolute Q DNA Digital PCR Master Mix (5×) (ThermoFisher Scientific), 0.45 μL of 20× TaqMan‐MGB‐FAM probe assay (ThermoFisher Scientific), 0.45 μL of 20× TaqMan‐MGB‐VIC probe assay (ThermoFisher Scientific), 0.45 μL of 20× TaqMan‐MGB‐ABY probe assay (ThermoFisher Scientific), 5 μL of cDNA, and 0.85 μL of nuclease‐free water (Qiagen) were mixed to obtain a 9 μL final volume of reaction mix. Each reaction mix was loaded in the Absolute Q MAP16 plate (ThermoFisher Scientific) and 15 μL of Absolute Q Isolation Buffer (ThermoFisher Scientific) was added. The thermocycling conditions were the following: 95°C for 8′, 43 cycles at 90°C for 15″, 60° for 1′, followed by a final extension step at 60°C for 2′.

### 2.7 Statistical Analysis

The statistical analysis has been performed using the GraphPad Prism software (version 10.0.2). More precisely, unpaired two‐tailed *t*‐test was used for the comparison of two specific treatment groups. Finally, to compare the data obtained between the three in vitro cell models tested, the Two‐way ANOVA with Tukey's post hoc test was also performed. Statistical significance was accepted at a probability level *p* < 0.05.

## 3 Results

### 3.1 Dimensional and Morphological Comparison Between K562, LAMA84, and KCL22 Cell Lines Following TKIs Treatment

**Figure 2:**
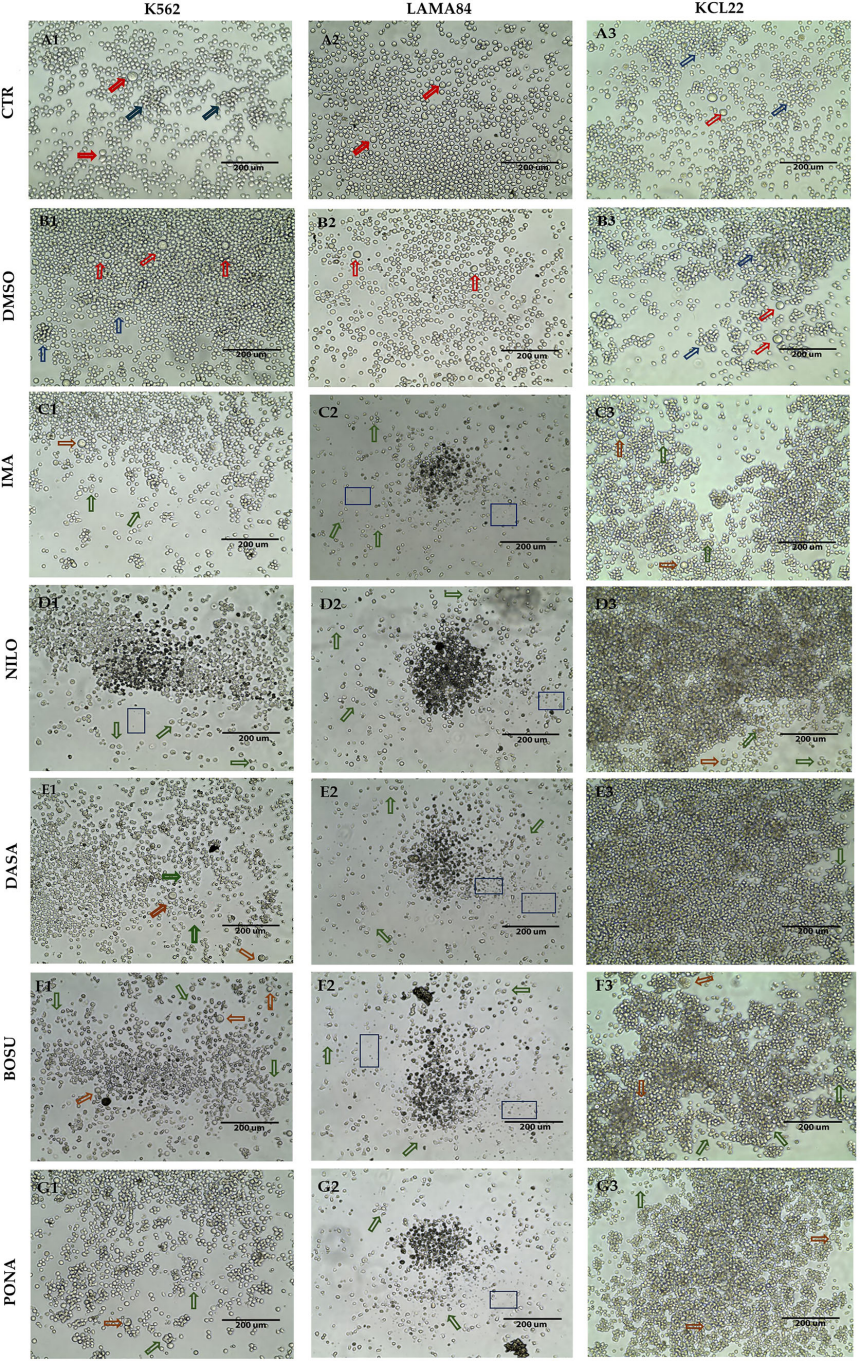
Panel of images illustrating the seven different treatment conditions of K562, LAMA84, and KCL22 cell lines obtained using the Invitrogen EVOS XL Core Imaging System with a 20× zoom. Areas of the well that best showed the cellular conditions were selected (including color, shape, size, and the formation of dead cell clusters). Panel A1, A2, A3: Control at 20× zoom in the three cell lines; Panel B1, B2, B3: DMSO at 20X zoom in the three cell lines; Panel C1, C2, C3: imatinib at 20× zoom in the three cell lines; Panel D1, D2, D3: nilotinib at 20× zoom in the three cell lines; Panel E1, E2, E3: dasatinib at 20× zoom in the three cell lines; Panel F1, F2, F3: bosutinib at 20× zoom in the three cell lines; Panel G1, G2, G3: ponatinib at 20× zoom in the three cell lines. Red arrows indicate significantly larger sized cells; blue arrow indicate cell clusters; orange arrows indicate larger sized cells; green arrows indicate irregularly shaped cells; blue boxes highlight apoptotic bodies. (CTR, control; DMSO, dimethylsulfoxide; IMA, imatinib; NILO, nilotinib; DASA, dasatinib; BOSU, bosutinib; PONA, ponatinib).

Images at 4x reveal cell positions and density within the well (Supporting Information S1: Figure S1), while images at 20x aid in estimating distinct differences following treatments across the three models (Figure 2). Comparing untreated (CTR) and DMSO‐treated cells, no differences are observed, confirming that DMSO does not trigger cytotoxicity when properly diluted. In both scenarios, cells tend to arrange in the center of the well forming high density areas, particularly in the case of K562 and KCL22 (Supporting Information S1: Figure S1). In all cases, cells exhibit an intact cell membrane, the absence of cytoplasmic granules, and a round morphology, which appears more heterogeneous in the case of LAMA84. The models show comparable cell sizes together with the presence of cells that are significantly larger than the surroundings (indicated with red arrows in Figure 2). Clusters formation is noted in both KCL22 and K562 lines, although the size is smaller in the case of the latter (indicated with blue arrows in Figure 2).

Upon TKIs treatment a strong reduction in cell numbers is visible in all cell lines, along with the presence of dead cells and apoptotic bodies. Nilotinib, dasatinib, and bosutinib seem to have a major impact on cell state, whereas imatinib appears to have less impact. In the case of LAMA84, however, all five TKIs seem to have a strong effect on the cells, with no evident differences between them. Additionally, even after being treated with TKIs, KCL22 cells tend to aggregate, resulting in a region with a higher cell density. However, a change in the cellular phenotype was noticed, marked by smaller cells and darker pigmentation, even though they are abundant in numbers.

In some instances, larger‐sized cells (indicated by orange arrows), irregularly shaped cells (highlighted with green arrows), and apoptotic bodies (marked with blue boxes) are observable.

Concerning the dimensional and morphological analysis, the average results following each treatment in the three cell lines are reported in Table 2. In general, all TKIs are able to reduce the average cell area and diameter in the three cell lines, with a lesser impact observed in the case of imatinib. For both CTR and DMSO, statistically significant differences are obtained when comparing K562 and KCL22 (*p* = 0.0449 and *p* = 0.0320, respectively). Nilotinib reveals significant differences when comparing K562 and LAMA84 (*p* = 0.0201) and LAMA84 and KCL22 (*p* = 0.0489). Dasatinib displays variations in the comparison of K562 vs. KCL222 (*p* = 0.0223). Lastly, for ponatinib, differences are observed in the cases of K562 vs. LAMA84 (*p* = 0.0233) and K562 vs. KCL22 (*p* = 0.0361). Cells of larger sizes (greater than 50% of other observed cells) have been excluded from the statistical analyses, which, in the case of controls and DMSO, are likely comparable to cells in imminent division, and in the case of TKIs, to cells in pre‐apoptosis.

**Table 2:** The average area, diameter, and circularity resulting from each treatment in the three cell lines utilized. Values that have shown statistically significant differences during comparison are highlighted in green. (BOSU, bosutinib; CTR, control; DASA, dasatinib; DMSO, dimethylsulfoxide; IMA, imatinib; NILO, nilotinib; PONA, ponatinib; TKIs, tyrosine kinase inhibitors). (Two Way ANOVA).

		K562	LAMA84	KCL22	*p*‐value of significant comparisons
CTR	Average area (μm²)	163.38	157.82	145.52	*p* = 0.0449
	Average diameter (μm)	14.43	14.2	13.62	
	Average circularity	0.36	0.48	0.49	
DMSO	Area (μm²)	151.44	136.05	133.69	*p* = 0.0320
	Diameter (μm)	13.89	13.16	13.05	
	Circularity	0.49	0.48	0.43	
IMA	Area (μm²)	105.29	86.22	103.88	
	Diameter (μm)	11.58	10.48	11.5	
	Circularity	0.39	0.51	0.3	
NILO	Area (μm²)	56.44	87.59	63.95	K562 vs. LAMA84, *p* = 0.0201LAMA84 vs. KCL22, *p* = 0.0489
	Diameter (μm)	8.47	10.56	9.03	
	Circularity	0.57	0.48	0.6	
DASA	Area (μm²)	100.37	78.04	70.47	K562 vs. KCL22, *p* = 0.0223
	Diameter (μm)	1.31	9.97	9.48	
	Circularity	0.643	0.46	0.8	
BOSU	Area (μm²)	109.02	91.64	89.49	
	Diameter (μm)	11.78	10.81	10.68	
	Circularity	0.42	0.39	0.51	
PONA	Area (μm²)	105.76	75.78	80.13	K562 vs. KCL22, *p* = 0.0361
	Diameter (μm)	11.61	9.82	10.1	
	Circularity	0.49	0.53	0.58	

In terms of circularity, there are no statistically significant differences among the investigated models under any condition.

### 3.2 Cell Viability Comparison Between K562, LAMA84, and KCL22 Cell Lines Following TKIs Treatment

Cell viability was evaluated by MTT assay in which MTT reagent is converted to a purple formazan product via mitochondrial dehydrogenases in living cells.

The MTT viability assay results, expressed as cell viability percentages, revealed a notable decrease in all three cell lines treated with TKIs compared to controls. Specifically, it's noteworthy that no statistically significant differences are observed between the three control groups nor between the TKIs groups (Supporting Information S1: Figure S2).

**Figure 3:**
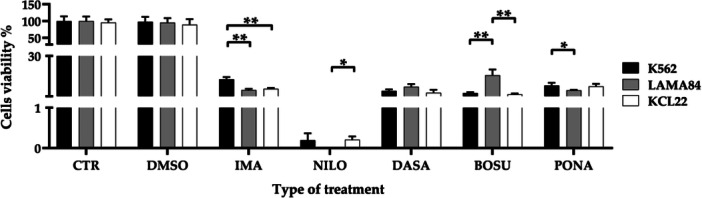
Cells viability (%) measured by MTT assay in the three cell lines considered, K562, LAMA84, and KCL22, representative of the comparison across individual treatment conditions (CTR, control; DMSO, dimethylsulfoxide; IMA, imatinib; NILO, nilotinib; DASA, dasatinib; BOSU, bosutinib; PONA, ponatinib) (Two‐way ANOVA with Tukey's post hoc test).

Further comparisons were conducted within each specific treatment condition resulting in many statistical differences (Figure 3). No significant differences were detectable between the control and DMSO groups. However, in the imatinib‐treated groups, differences emerged in the comparisons between K562 and LAMA84 (*p* = 0.0017) and K562 and KCL22 (*p* = 0.0025). Within the nilotinib‐treated groups, a significant difference is noted in the comparison between LAMA84 and KCL22 (*p* = 0.0114). Similarly, the bosutinib‐treated groups displayed substantial differences in the comparisons between K562 and LAMA84 (*p* = 0.0057) and LAMA84 and KCL22 (*p* = 0.0044). Finally, in the ponatinib‐treated groups, significant values were identified in the comparison between K562 and LAMA84 (*p* = 0.0375).

### 3.3 Metabolic Activity Comparison Between K562, LAMA84, and KCL22 Cell Lines Following TKIs Treatment

The number of metabolically active cells was evaluated via CCK8 assay which relies on the measurement of the absorbance, directly proportional to the number of cells with enzymatic activity. Briefly, CCK‐8 assay uses water‐soluble WST‐8 for sensitive colorimetric determination of active cell numbers, where cell dehydrogenases reduce WST‐8 to soluble formazan proportional to the number of metabolically active cells. In all cell lines treatment with TKIs reveals reduced cells when compared to controls. Notably, among the three control groups, no statistically significant differences were observed. Whereas, in DMSO‐treated groups, a significant decrease was observed in LAMA84 vs. K562 and KCL22 (*p* = 0.0086 and *p* = 0.0262, respectively). Among the TKIs treatment groups, significant differences emerged between all cell lines (K562 vs. KCL22, *p* = 0.0013; KCL22 vs. LAMA84, *p* = 0.0022; K562 vs. LAMA84, *p* = 0.0471) (Supporting Information S1: Figure S3).

**Figure 4:**
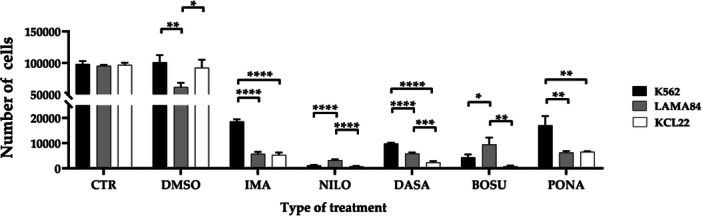
Number of cells measured by CCK8 assay in the three cell lines considered, K562, LAMA84 and KCL22, representative of the comparison across individual treatment conditions (CTR, control; DMSO, dimethylsulfoxide; IMA, imatinib; NILO, nilotinib; DASA, dasatinib; BOSU, bosutinib; PONA, ponatinib) (Two‐way ANOVA with Tukey's post hoc test).

Moreover, when considering the single TKI, the trend described above is confirmed (Figure 4). In the imatinib‐treated groups, statistically significant differences were observed in comparisons between K562 and LAMA84 (*p* < 0.0001) and K562 and KCL22 (*p* < 0.0001). In the nilotinib‐treated groups, significant differences were observed in comparisons K562 and LAMA84 (*p* < 0.0001) and LAMA84 and KCL22 (*p* < 0.0001). Dasatinib‐treated groups exhibit statistically significant differences in all comparisons: K562 vs. LAMA84 (*p* < 0.0001), K562 vs. KCL22 (*p* < 0.0001), and LAMA84 vs. KCL22 (*p* = 0.0001). Similarly, bosutinib‐treated groups showed significant differences in every comparison: K562 vs. LAMA84 (*p* = 0.0217) and LAMA84 vs. KCL22 (*p* = 0.0017). Finally, in the case of ponatinib, statistically significant differences were observed when comparing K562 with LAMA84 (*p* = 0.0020) and K562 with KCL22 (*p* = 0.0023).

### 3.4 Comparison of Extracellular Glutamate Concentration in K562, LAMA84, and KCL22 Cell Lines Following TKIs Treatment

Then, we evaluated the intake of glutamate resulting from the individual treatments in the three investigated cell lines, as it represents an altered pathway in the pathogenesis of CML. All results express the concentration of extracellular glutamate present in the culture medium and must therefore be interpreted as inversely proportional to aminoacid intake.

Evaluating the results obtained from the CTR and TKIs groups reveals substantial differences (Supporting Information S1: Figure S4). Considering untreated cells, extracellular glutamate decreased in both K652 and KCL22 cell lines compared to LAMA84 (K562 vs. LAMA84, *p* = 0.0010; K562 vs. KCL22, *p* < 0.0001; LAMA84 vs. KCL22, *p* < 0.0001). Moreover, among the TKIs treatment groups, the maximum significant valence (*p* < 0.0001) was achieved in all comparisons performed.

**Figure 5:**
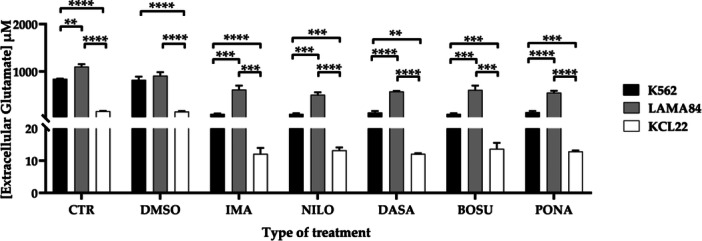
Extracellular glutamate concentration (μM) in the three cell lines considered, K562, LAMA84, and KCL22, representative of the comparison across individual treatment conditions (CTR, control; DMSO, dimethylsulfoxide; IMA: imatinib; NILO, nilotinib; DASA, dasatinib; BOSU, bosutinib; PONA, ponatinib) (Two‐way ANOVA with Tukey's post hoc test).

When considering individual treatments (Figure 5), maximum statistical significance was noted under DMSO conditions, when comparing both LAMA84 vs. KCL22, and K562 vs. KCL22 (*p* < 0.0001 for both). For imatinib, significant differences were found in all comparisons: K562 vs. LAMA84 (*p* = 0.0004), LAMA84 vs. KCL22 (*p* = 0.0002), and K562 vs. KCL22 (*p* < 0.0001). Similarly, with nilotinib, differences were observed in each comparison: K562 vs. LAMA84 (*p* = 0.0002), LAMA84 vs. KCL22 (*p* < 0.0001), and K562 vs. KCL22 (*p* = 0.0003). With bosutinib, statistical significance was obtained as follows: K562 vs. LAMA84 (*p* = 0.0007), LAMA84 vs. KCL22 (*p* = 0.0003), and K562 vs. KCL22 (*p* = 0.0003). Finally, ponatinib also showed significance in each comparison: K562 vs. LAMA84 (*p* < 0.0001), LAMA84 vs. KCL22 (*p* < 0.0001), and K562 vs. KCL22 (*p* = 0.0005).

### 3.5 *BCR::ABL1, CD33* and *CD11b* Transcripts Expression Comparison in K562, LAMA84, and KCL22 Cell Lines Following TKIs Treatment

The expression of the disease hallmark *BCR::ABL1*, the monocytic marker *CD33*, and the neutrophilic marker *CD11b* in the three cell lines studied was analyzed using dPCR. This analysis involved normalizing their expression levels relative to the housekeeping gene *GAPDH*.

Starting with *BCR::ABL1*, a decrease was evident in all three models when comparing the control and TKIs groups, as expected (Supporting Information S1: Figure S5). Additionally, among the three control groups, a statistically significant difference was noted when comparing K562 and KCL22 (*p* = 0.0139) and LAMA84 and KCL22 (*p* = 0.0277). Similarly, among the treatment groups with TKIs, statistically significant values were obtained in the comparisons of K562 vs. LAMA84 (*p* = 0.0038) and K562 vs. KCL22 (*p* = 0.0017).

**Figure 6:**
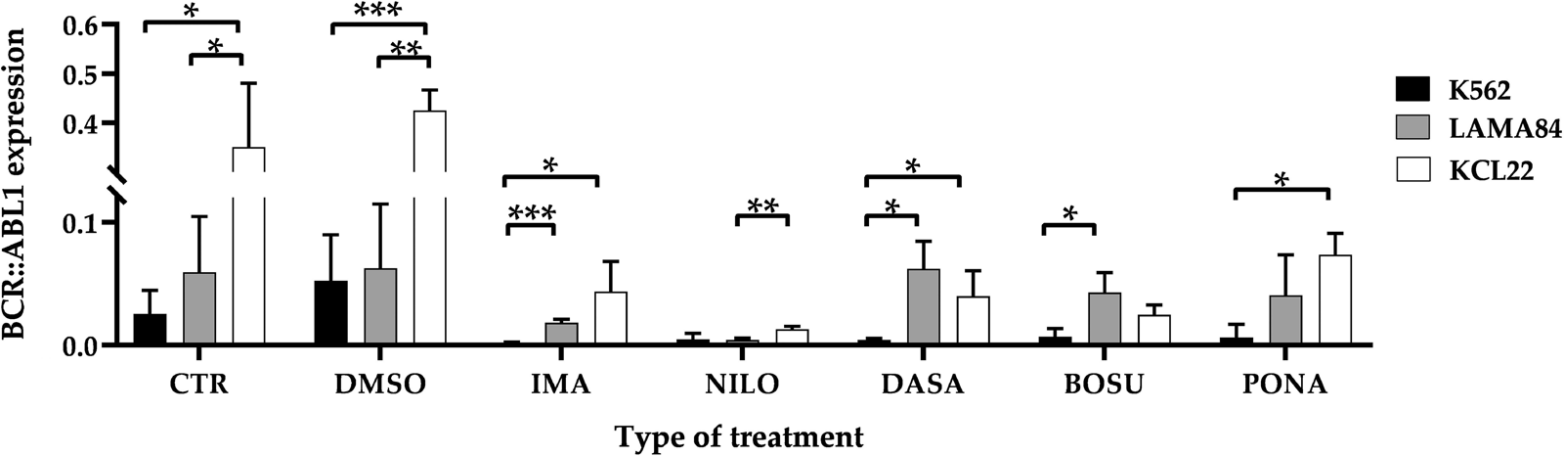
Expression of BCR::ABL1 transcript (normalised for GAPDH) in the three cell lines considered, K562, LAMA84 and KCL22, representative of the comparison across individual treatment conditions (CTR, control; DMSO, dimethylsulfoxide; IMA, imatinib; NILO, nilotinib; DASA, dasatinib; BOSU, bosutinib; PONA, ponatinib) (Two‐way ANOVA with Tukey's post hoc test).

Focusing on individual treatments (Figure 6), statistically significant differences were evident between different cell lines. For DMSO, significant distinctions were reported in LAMA84 vs. KCL22 (*p* = 0.0048) and K562 vs. KCL22 (*p* = 0.0006). In the imatinib‐treated cells, a significant difference is reported in K562 vs. LAMA84 (*p* = 0.0007) and K562 vs. KCL22 (*p* = 0.0399). Among the nilotinib‐treated groups, there is a substantial difference in the LAMA84 vs. KCL22 comparison (*p* = 0.0043). In the dasatinib‐treated cells, variations are observed when comparing K562 and LAMA84 (*p* = 0.0159) and K562 and KCL22 (*p* = 0.0407). Finally, in bosutinib‐treated cells, significance is only found in the case of K562 vs. LAMA84 (*p* = 0.0224), and in ponatinib‐treated cells in K562 vs. KCL22 (*p* = 0.0144). Examining the second transcript analyzed, the monocytic differentiation marker *CD33*, a statistically significant difference was evident in the control groups when comparing K562 vs. LAMA84 (*p* = 0.0034) and LAMA84 vs. KCL22 (*p* = 0.0016). In the TKIs groups, a significant difference is observed only in the comparison of LAMA84 vs KCL22 (*p* = 0.0088) (Supporting Information S1: Figure S6).

**Figure 7:**
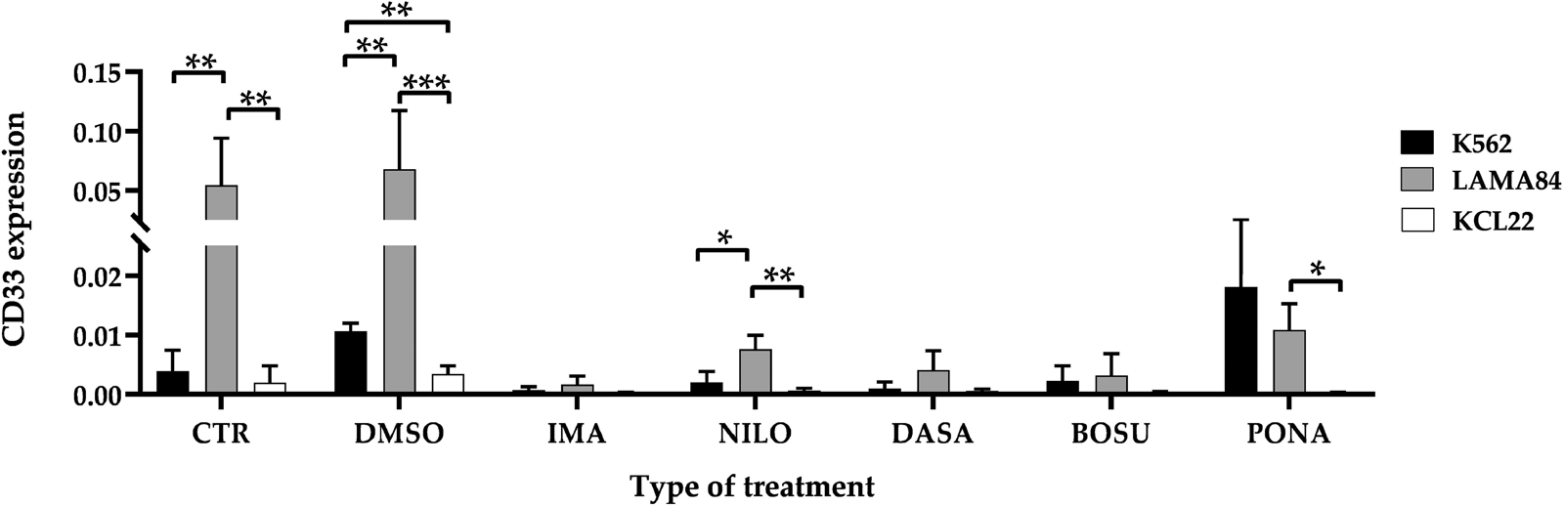
Expression of CD33 transcript (normalized for GAPDH) in the three cell lines considered, K562, LAMA84, and KCL22, representative of the comparison across individual treatment conditions (CTR, control; DMSO, dimethylsulfoxide; IMA, imatinib; NILO, nilotinib; DASA, dasatinib; BOSU, bosutinib; PONA, ponatinib) (Two‐way ANOVA with Tukey's post hoc test).

Analyzing individual treatments, significant differences emerge. For DMSO treatment, differences were evident between K562 and LAMA84 (*p* = 0.0075), LAMA84 and KCL22 (*p* = 0.0005), and K562 and KCL22 (*p* = 0.0093). Subsequently, statistically significant differences arose with nilotinib treatment when comparing K562 vs. LAMA84 (*p* = 0.0316) and LAMA84 vs. KCL22 (*p* = 0.0070), and with ponatinib treatment, particularly comparing LAMA84 vs. KCL22 (*p* = 0.0432) (Figure 7).

Finally, we evaluated the expression of the neutrophil differentiation marker *CD11b*. Comparison across the three investigated cell lines reveals statistically significant differences between the control groups in K562 vs. LAMA84 (*p* = 0.0014), and LAMA84 vs. KCL22 (*p* = 0.0032). For TKIs groups, significance arose when comparing K562 vs. LAMA84 (*p* = 0.0275) and LAMA84 vs. KCL22 (*p* = 0.0028) (Supporting Information S1: Figure S7).

**Figure 8:**
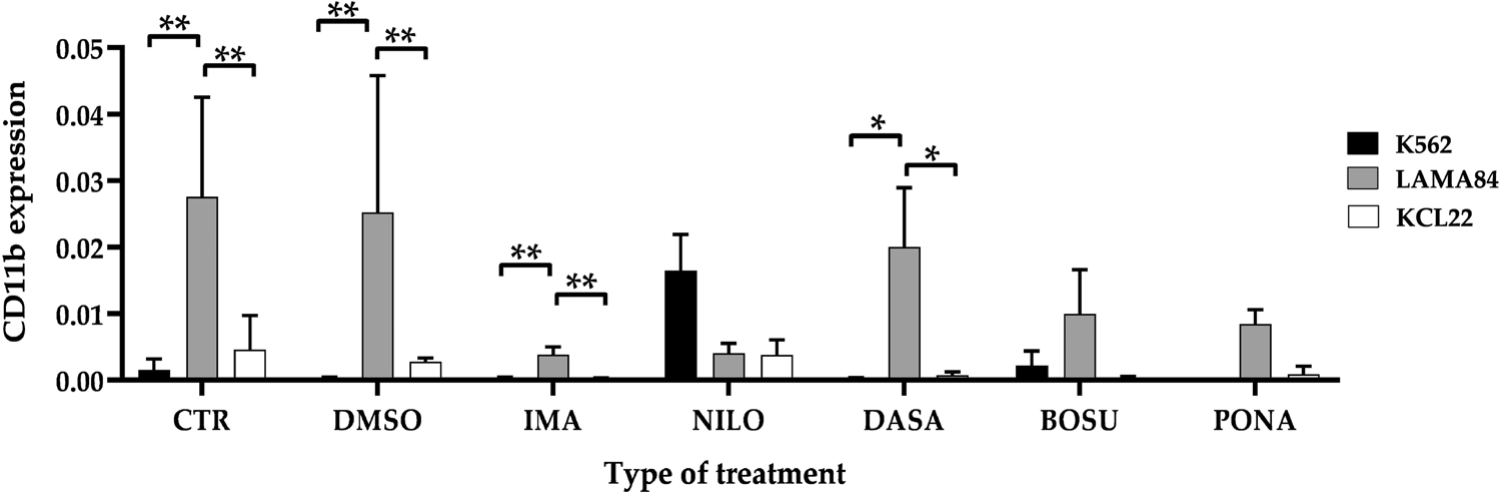
Expression of CD11b transcript (normalized for GAPDH) in the three cell lines considered, K562, LAMA84, and KCL22, representative of the comparison across individual treatment conditions (CTR, control; DMSO, dimethylsulfoxide; IMA, imatinib; NILO, nilotinib; DASA, dasatinib; BOSU, bosutinib; PONA, ponatinib) (Two‐way ANOVA with Tukey's post hoc test).

Focusing on individual treatments, significance was observed under DMSO conditions for K562 vs. LAMA84 (*p* = 0.0056) and LAMA84 vs. KCL22 (*p* = 0.0041). Regarding single TKIs, differences were noted with imatinib between K562 vs. LAMA84 (*p* = 0.0054) and K562 vs. KCL22 (*p* = 0.0054). Statistically significant results also emerged with dasatinib, particularly in K562 vs. LAMA84 (*p* = 0.0179) and LAMA84 vs. KCL22 (*p* = 0.0198), and with ponatinib, specifically in LAMA84 vs. KCL22 (*p* = 0.0058) (Figure 8).

### 3.6 Evaluation of the Effects of Asciminib in Terms of Dimension and Morphology, Viability, Metabolic Activity, Extracellular Glutamate Concentration, and the Expression of *BCR::ABL1*, *CD33*, and *CD11b* Transcripts in K562, LAMA84, and KCL22 Cell Lines After Individual Treatments

Given its distinct mechanism of action, the following paragraph summarizes the outcomes of asciminib treatment across the three selected cell lines.

**Figure 9:**
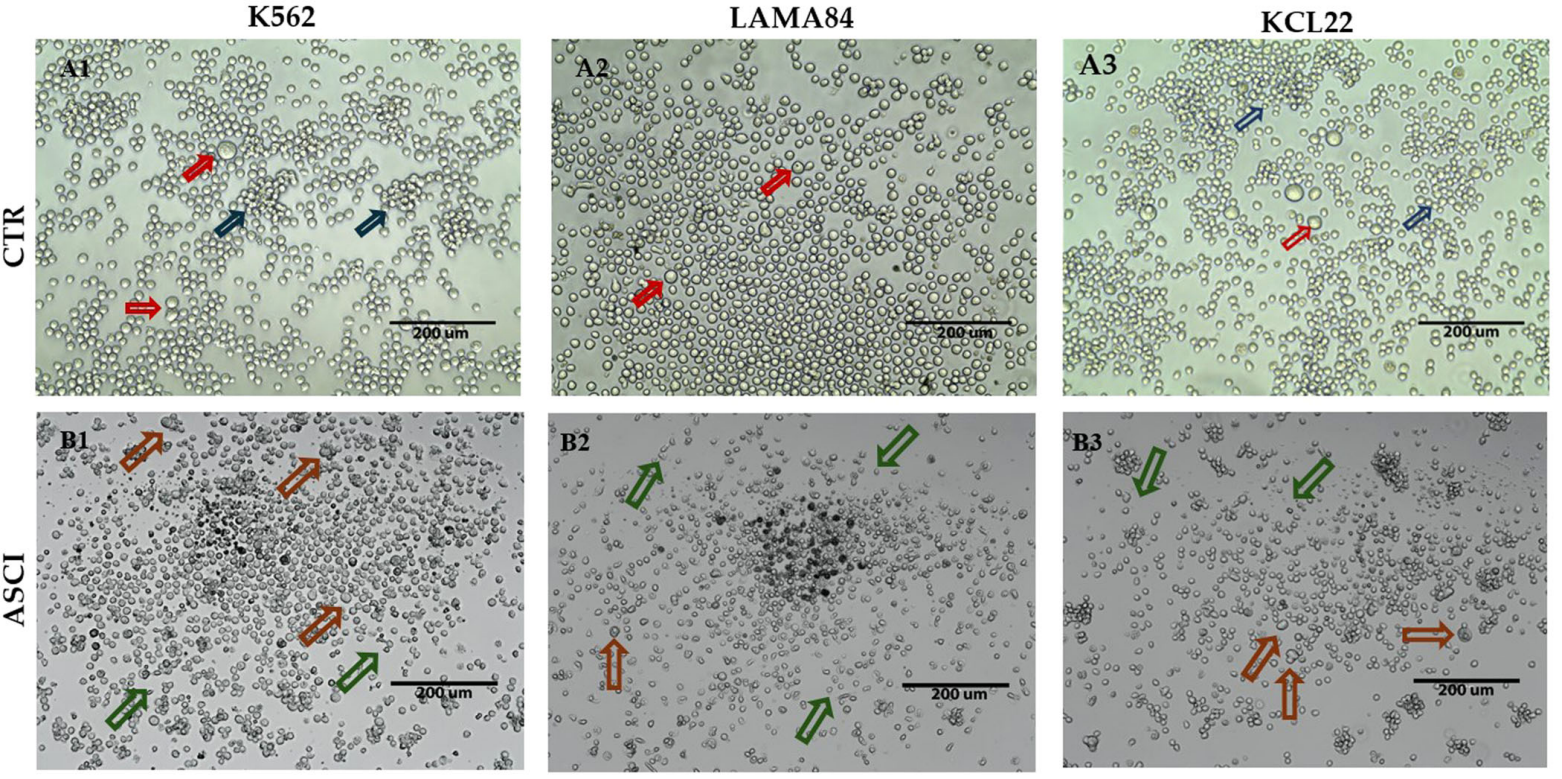
Panel of images illustrating asciminib treatment of K562, LAMA84, and KCL22 cell lines obtained using the Invitrogen EVOS XL Core Imaging System with a 20× zoom. Areas of the well that best showed the cellular conditions were selected (including color, shape, size, and the formation of dead cell clusters). Panel A1, A2, A3: Control at 20× zoom in the three cell lines; Panel B1, B2, B3: asciminib at 20× zoom in the three cell lines. Red arrows indicate significantly larger sized cells; blue arrow indicate cell clusters; orange arrows indicate larger sized cells; green arrows indicate irregularly shaped cells. (CTR, control; ASCI, asciminib).

When evaluating the images under optical microscopy, the 4× magnification revealed that treatment with asciminib, compared to untreated cells, led to a reduction in the number of cells that did not tend to arrange themselves at the center of the well in all three cell lines tested (Supporting Information S1: Figure S8). At 20× magnification (Figure 9), it became evident that asciminib was capable of affecting the cellular status, resulting in altered morphology (as indicated by green arrows), a likely reduction in cell size, the appearance of larger cells, likely undergoing imminent lysis (highlighted by orange arrows), and cells resembling dead cells, as indicated by their dark pigmentation.

Concerning the dimensional and morphological analysis, the average results following each treatment in the three cell lines are reported in Table 3. No differences emerged in terms of average cell area, diameter, or circularity across the three cell lines when treated with asciminib.

**Table 3:** The average area, diameter, and circularity resulting from asciminib treatment in the three cell lines utilized. Values that have shown statistically significant differences during comparison are highlighted in green. (ASCI, asciminib; CTR, control). (Two Way ANOVA).

		K562	LAMA84	KCL22	*p*‐value of significant comparisons
CTR	Average area (μm²)	163.38	157.82	145.52	*p* = 0.0449
	Average diameter (μm)	14.43	14.2	13.62	
	Average circularity	0.36	0.48	0.49	
ASCI	Area (μm²)	116.83	101.64	106.38	
	Diameter (μm)	12.2	11.88	11.64	
	Circularity	0.84	0.82	0.64	

**Figure 10:**
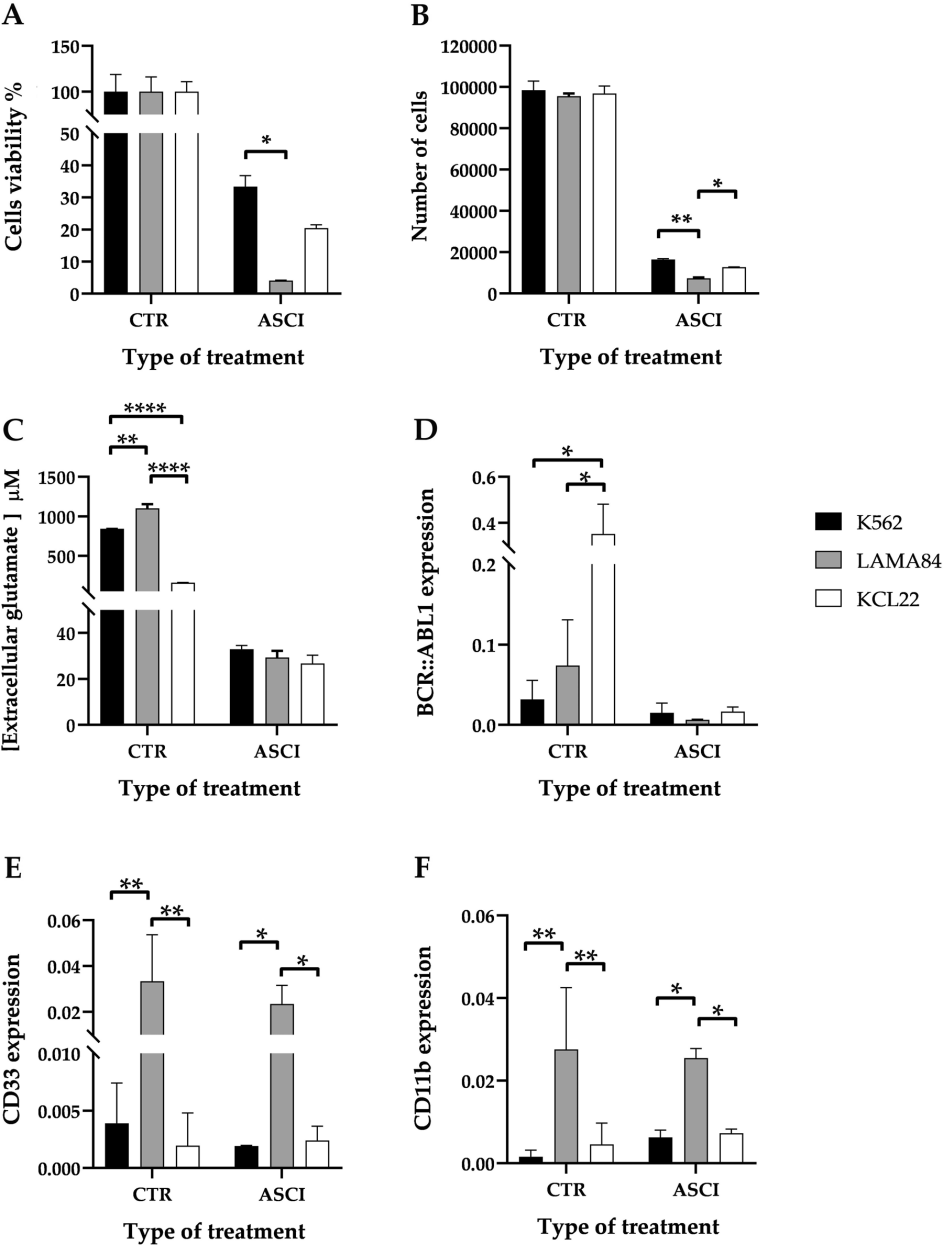
Cell viability (%) measured by MTT assay (A), number of cells measured by CCK8 assay (B), extracellular glutamate concentration (μM) (C) and expression of BCR::ABL1 (D), CD33 (E) and CD11b (F) transcripts all normalized for GAPDH in the three cell lines considered, K562, LAMA84 and KCL22, following asciminib treatment. (CTR, control; ASCI, asciminib) (Two‐way ANOVA with Tukey's post hoc test).

When cell viability was assessed using the MTT test (Figure 10A), treatment with asciminib reduced cell viability compared to untreated cells. Notably, a statistically significant difference was observed between the K562 and LAMA84 cell lines (*p* = 0.0211), while a nonsignificant decreasing trend was also evident when comparing KCL22 to LAMA84.

These findings were corroborated by the analysis of metabolic activity using the CCK8 assay (Figure 10B), which showed a reduction of cell number in treated cells compared to controls. Significant differences were particularly evident when comparing LAMA84 to both K562 and KCL22 (LAMA84 vs. K562, *p* = 0.0015; LAMA84 vs. KCL22, *p* = 0.0492).

The evaluation of extracellular glutamate concentration demonstrated that asciminib induces glutamate uptake in all analyzed models, with no statistically significant differences observed between them (Figure 10C).

Analyzing the expression of the disease hallmark *BCR::ABL1* transcript through dPCR, the treatment with asciminib led to a reduction across all three in vitro models compared to controls. No statistically significant differences were observed among the treated cells across the three models (Figure 10D).

Regarding the expression of the monocytic marker CD33, the STAMP inhibitor similarly induced a reduction when comparing treated cells to the untreated ones. Statistically significant differences emerged when comparing LAMA84 to both K562 and KCL22 (LAMA84 vs. K562, *p* = 0.0210; LAMA84 vs. KCL22, *p* = 0.0237) (Figure 10E).

Finally, the evaluation of the neutrophilic differentiation marker C11b also revealed a decrease following asciminib treatment relative to untreated models. Notably, statistically significant differences were observed between LAMA84 and both K562 and KCL22 (LAMA84 vs. K562, *p* = 0.0127; LAMA84 vs. KCL22, *p* = 0.0273) (Figure 10F).

## 4 Discussion

Chronic Myeloid Leukemia (CML) is a myeloproliferative disorder originating from the hematopoietic stem cell compartment. Its etiopathogenesis is attributed to the balanced translocation t(9;22), involving the BCR gene (ch.22) and the ABL1 gene (ch.9), leading to the formation of the BCR::ABL1 oncogene and its corresponding and constitutively active protein (Chereda and Melo 2015; Khoury et al. 2022; Quintás‐Cardama and Cortes 2009). Targeted therapy with Tyrosine Kinase Inhibitors (TKIs) like imatinib, dasatinib, nilotinib, bosutinib, and ponatinib has transformed CML treatment since the early 2000s (Cortes et al. 2018; Lipton et al. 2016; Radich et al. 2012; Saglio et al. 2010), resulting in high response rates and in the establishment of numerous drug discontinuation trials (Abruzzese et al. 2023; Chereda and Melo 2015; Malagola et al. 2021, 2024). Before their introduction, patients with CML had an average overall survival of 3 years (Chereda and Melo 2015). Currently, life expectancy is comparable to that of a healthy population. For patients resistant to multiple TKIs, in 2021 the FDA approved the first‐in‐class allosteric Specifically Targeting the ABL Myristoyl Pocket (STAMP) inhibitor, asciminib. It has shown superior efficacy compared to bosutinib in the phase III ASCEMBL trial and is under investigation as a first‐line therapy in the ASC4FIRST trial (Cortes et al. 2022; Réa et al. 2021). However, challenges such as side effects, noncompliance, and TKIs resistance persist, highlighting unmet clinical needs. Establishing reliable preclinical models, especially in vitro, is crucial for translating laboratory findings into clinical practice. In this context, in vitro models play a crucial role, facilitating cancer research by aiding in the identification of carcinogens, drug development, and understanding molecular mechanisms (Drexler et al. 1999; Greshock et al. 2007; Holliday and Speirs 2011; Katt et al. 2016). Ph+ cell lines, reported in Table 1, offer valuable models for studying various leukemic states beyond chronic phase CML (Drexler et al. 1999). In scientific experimentation, it's common practice to employ a single in vitro model as the primary biological representation of a pathology. Although evidence for model interchangeability is lacking, exploring diverse in vitro approaches improves our understanding of pathology and treatment, aiding in personalized therapy design. In the specific field of CML, to our knowledge, only one study has identified differences in protein expression levels among three CML cell lines treated with imatinib, but other investigations with the same aim are not found in the literature (Fontana et al. 2007). Our study has demonstrated significant differences among the three most used CML models in research, the K562, LAMA84, and KCL22 cell lines.

The intermodel comparison via optical microscopy, even under control conditions, has revealed significant dimensional differences in terms of average area between K562 and KCL22, both exhibiting similar morphologies and propensity to form clusters, whereas LAMA84 display greater diversity despite no discernible difference in circularity. All treatments tested exhibit an impact on cell numbers and status, leading to the formation of apoptotic bodies, cytoplasmic granules, and membrane irregularities. However, their effects vary across cell lines. Notably, the average cell area decreases differently depending on the TKI in both K562 and KCL22, while showing a more uniform decrease in LAMA84. Differences between the three cell lines are confirmed with nilotinib, and ponatinib, while in the case of dasatinib, only comparing LAMA84 and KCL22. However, asciminib does not result in statistically significant differences in these parameters.

These variations are further identified by viability and metabolic activity tests, yielding comparable results. In both cases, although starting from control conditions showing no significant differences among the three lines, differences between models are observed in various treatment conditions. Firstly, considering TKIs as a single treatment strategy, no differences in viability emerge among the models; whereas regarding metabolic activity, KCL22 cells exhibit the lowest levels, intermediate in LAMA84, and highest in K562. Delving into individual treatments, both analyses highlight nilotinib's major impact on all three lines, with differences between models in both vitality and metabolic activity. Conversely, imatinib and ponatinib appear to have the least impact on both parameters, although differences between models also emerge in this case. The results are attributed to imatinib's lower potency as a first‐generation inhibitor or ponatinib's specificity for the T315I mutation, which was not present in the tested cell lines (Cortes et al. 2012; Hochhaus et al. 2013). Dasatinib and bosutinib yield intermediate results with further differences among the selected cell lines. Cell viability results do not completely align with those reported by Tusa et al., who observed a progressive reduction in cell viability from a first‐generation TKI to second‐generation and third generation through estimation with Trypan Blue (Tusa et al. 2020). Additionally, they reported similar results among the same three cell lines tested by us, suggesting the interchangeability of one model over another in defining TKIs effects. Similarly, treatment with asciminib reduced both cell viability and metabolic activity, as already widely reported in the literature (Manley et al. 2020; Wylie et al. 2017). Particularly, a significant difference in cell viability was observed between K562 and LAMA84, with LAMA84 showing a greater reduction. Differences in metabolic activity were also found between LAMA84 and both K562 and KCL22. These results show that asciminib affects cell lines differently, with LAMA84 being more sensitive than K562 and KCL22. This difference between models hasn't been reported before, except in comparisons between TKI‐sensitive lines and those with specific mutations (Okabe et al. 2024a; Okamoto et al. 2024).

Moving on to the analysis of the glutamate pathway, in the context of CML it is known to be involved in disease progression and in the development of resistance to TKIs (Koda et al. 2023; Mostazo et al. 2020). Furthermore, a study conducted by Poteti et al. have already described the effect of glutaminase inhibition in the setting of CML and confirmed the potential therapeutic effect of this latter (Poteti et al. 2021). We opted for investigating the level of the intake of a specific aminoacid to gain insight into the same pathway by evaluating how the cell lines react to the treatments' stimuli interacting with the extracellular environment. Differences are again observed among the three models. When untreated cells are considered, extracellular glutamate levels vary between models, with the KCL22 line having significantly lower levels. Upon TKIs and STAMP inhibitor treatments, all models display higher glutamate intake, with substantial differences between the three models based on the single therapeutic strategy. These results suggest a general correlation between increased glutamate retention and reduced metabolic activity and viability observed in the cell lines. Indeed, glutamate retention, as already observed in non‐leukemic in vitro models such as hepatocellular carcinoma, neuronal and ocular pathologies, and embryonic stem cells, could lead to a cytotoxic effect associated with increased oxidative stress and excessive glutamate receptor activation (Abd Jalil et al. 2017; Kritis et al. 2015; Seol et al. 2016).

Turning to the evaluation of *BCR::ABL1* transcript, comparing values in control groups reveals a higher value in KCL22 compared to the other two cell lines. This observation can be attributed to the fact that the KCL22 cell line has a faster doubling time (24 h) compared to K562 and LAMA84 (35 h and 50 h, respectively) (https://www.Dsmz.de/Collection/Catalogue/Details/Culture/ACC-168; https://www.Dsmz.de/Collection/Catalogue/Details/Culture/ACC-519; https://www.Sigmaaldrich.Com/IT/It/Product/Sigma/Cb_89121407). Moving on to individual treatments, differences are evident across models, although both TKIs and asciminib are effective in reducing the specific target for which they were formulated. It is noteworthy that in all three models, the most pronounced effect is observed with nilotinib and asciminib, although in the case of asciminib, no statistically significant differences are found between the models.

Finally, analyses of the monocytic *CD33* and neutrophilic *CD11b* transcripts confirm the existence of variances between the models (Olingy et al. 2022; Pillay et al. 2013). Results regarding untreated cells are quite consistent with data from The Human Protein Atlas research tool (https://www.Proteinatlas.Org/ENSG00000105383-CD33/Cell+line; https://www.Proteinatlas.Org/ENSG00000169896-ITGAM/Cell+line), where a higher baseline expression level of both transcripts is found in LAMA84, intermediate in K562, and lower in KCL22. However, considering the *CD11b* transcript, an intermediate value is found in KCL22 cells. Upon treatment with TKIs and STAMP inhibitor, a reduced expression of the *CD33* transcript is observed across all conditions. Specifically, for TKIs, the reduction is less pronounced and more comparable between K562 and LAMA84, while it is greater in KCL22. In contrast, asciminib treatment results in a more pronounced reduction in K562 and KCL22, with a lesser reduction observed in LAMA84. A possible cause of this discrepancy may again lie in the different doubling time between the models. Among all the treatments evaluated, only the K562 cell line treated with ponatinib exhibited an increase in transcript concentration compared to both the control and the other cell models. For the *CD11b* transcript, considering both TKIs and STAMP inhibitor treatment, the general trend of the three in vitro models is similar to that seen for *CD33*. However, unlike CD33, no substantial variation is found in K562 cells. The only exception is observable under nilotinib and asciminib treatments, which induce an increase in the transcript levels in K562 cells but not in the other two lines. In the case of asciminib, a slight increase in transcript levels is also observed in KCL22 compared to untreated cells. Overall, these analyses underscore intrinsic differences between the selected models, which are not commonly reported in the literature. These findings demonstrate that treatments with distinct mechanisms of action elicit differential responses among the used cell lines, emphasizing variations that may stem from the different origins of the models, as delineated in Table 1 and available from various biobanks (https://www.Coriell.Org/1/Browse/Biobanks; https://www.Dsmz.de/Collection/Catalogue/Human-and-Animal-Cell-Lines/Catalogue). Specifically, K562, LAMA84, and KCL22 originate from distinct lineages: erythrocytic, myelocytic, and ery‐megakaryocytic, respectively. Furthermore, the two most similar untreated lines (K562 and KCL22) both derive from pleural effusion, whereas LAMA84 originates from peripheral blood.

## 5 Conclusions

Our study underscores the importance of selecting appropriate in vitro models for preclinical research. Researchers must consider the cellular origin, genetic background, and phenotypic characteristics of each cell line when designing experiments as they reflect the inter‐patients' variability.

The results of the present study are not expected to be directly applied in clinical practice or clinical trials. Nevertheless, they could be an important starting point for preclinical research, which should therefore be based on several cell lines and strongly consider the differences between the used in vitro models when interpreting the results. Consequently, we will probably have an immediate impact on the in vitro and in vivo study phases and only later on the patients. In conclusion, our findings highlight the limitations of relying on a single cell line as a representative model for CML studies. Utilizing multiple cell lines and corroborating findings with ex vivo and in vivo models can enhance the robustness and translational relevance of preclinical research outcomes.

## Author Contributions

Conceptualization: A.C., B.X., S.B., S.M., and D.R. Data curation: A.C., S.M. and B.X. Formal analysis: A.C., B.X., S.B., S.M., L.G., A.L., and D.R. Funding acquisition: D.R. Investigation: A.C., B.X., R.M.F., E.L.M., S.C.G., F.T, F.R., and S.M. Methodology: A.C., and B.X. Project administration: S.B. Resources: M.F., M.M., E.M, G.M., V.C., E.A.B., V.R., and D.R. Software: A.C., L.G., and A.L. Supervision: S.B. Validation: A.C., B.X., S.B., S.M. Visualization: A.C., B.X., S.B., S.M. Writing – original draft preparation: A.C. Writing – review and editing: A.C, and S.B.

## Conflicts of Interest

The authors declare no conflicts of interest.

## Supporting information

Supporting information.

## Data Availability

The data that support the findings of this study are available from the corresponding author upon reasonable request.
