# Supplementary material for: Different In Vitro Models of Chronic Myeloid Leukemia Show Different Characteristics: Biological Replicates Are Not Biologically Equivalent
Source: Cell Biol Int. 2025 Mar 1;49(5):570–86. doi: 10.1002/cbin.70007 (PMC11994880; doi:10.1002/cbin.70007)
Supplement: Supplementary file 1 — Supporting information. [file CBIN-49-570-s001.docx]

**Supplementary Materials**

**Cell lines karyotype**

The cell lines used in the present manuscript presented differences both in terms of karyotype (KCL22: human hyperdiploid karyotype with 3.3% polyploidy - 51(47-51)<2n>X, -X, +1, +6, +8, +8, +14, +22, del(1)(p22), t(9;22)(q34;q11), add(17)(p12-13), i(21q), der(22)t(9;22)(q34;q11) – K562: human hypotriploid karyotype with sharp mode - 61-68<3n>XX, -X, -3, +7, -13, -18, +3mar, del(9)(p11/13), der(14)t(14;?)(p11;?), der(17)t(17;?)(p11/13;?), der(?18)t(15;?18)(q21;?q12), del(X)(p22) – LAMA84: human hypertriploid karyotype with 3.6% polyploidy - 73/74(69-77)<3n>XX, -X, +1, -2, +5, +6, +8, +13, -14, +17, +17, -18, +22, +mar, del(7)(p15), der(9)t(9;22)(q34;q11)x2, i(11q), add(13)(q33), del(17)(p12), der(22)t(9;22)(q34;q11)x4 - carries Ph (4 copies)) and in terms of immunophenotype (KCL22: CD3 -, CD4 +, CD13 +, CD14 (+), CD15 +, CD19 -, CD25 -, HLA-DR -. K562: CD3 -, CD14 -, CD15 +, CD19 -, CD33 +, CD71 +, CD235a +. LAMA84: CD3 -, CD11b +, CD13 +, CD15 -, CD19 -, CD33 +). Considering the more than forty known and stable CML cell lines, we are not able to identify a perfect biological duplicate (meaning two cell lines presenting the same karyotype and/or the same immunophenotype), so a direct correlation between these phenotypes and the sensitivity to TKIs is not evaluable.

**Figure S1:** Panel of images illustrating the seven different treatment conditions of K562, LAMA84, and KCL22 cell lines obtained using the Invitrogen^TM^ EVOS^TM^ XL Core Imaging System with a 4X zoom. **Panel A1, A2, A3**: Control at 4X zoom in the three cell lines; Panel B1, B2, B3: DMSO at 4X zoom in the three cell lines; Panel C1, C2, C3: imatinib at 4X zoom in the three cell lines; Panel D1, D2, D3: nilotinib at 4X zoom in the three cell lines; Panel E1, E2, E3: dasatinib at 4X zoom in the three cell lines; Panel F1, F2, F3: bosutinib at 4X zoom in the three cell lines; Panel F1, F2, F3: ponatinib at 4X zoom in the three cell lines. (CTR: control, DMSO: Dimethylsulfoxide, IMA: imatinib, NILO: nilotinib, DASA: dasatinib, BOSU: bosutinib, PONA: ponatinib).

**Figure S2:** Cells viability (%) measured by MTT assay in the three cell lines considered, K562, LAMA84 and KCL22, representative of the comparisons within the two treatment groups. (CTR: control, TKIs: Tyrosine Kinase Inhibitors) (Unpaired two-tailed t-test).

**Figure S3:** Number of cells measured by CCK8 assay in the three cell lines considered, K562, LAMA84 and KCL22, representative of the comparisons within the two treatment groups. (CTR: control, TKIs: Tyrosine Kinase Inhibitors) (Unpaired two-tailed t-test).

**Figure S4:** Extracellular glutamate concentration (μM) in the three cell lines considered, K562, LAMA84 and KCL22, representative of the comparisons within the two treatment groups. (CTR: control, TKIs: Tyrosine Kinase Inhibitors) (Unpaired two-tailed t-test).

**Figure S6:** Expression of CD33 transcript (normalised for GAPDH) in the three cell lines considered, K562, LAMA84 and KCL22, representative of the comparisons within the two treatment groups. (CTR: control, TKIs: Tyrosine Kinase Inhibitors) (Unpaired two-tailed t-test)

**Figure S5:** Expression of BCR::ABL1 transcript (normalised for GAPDH) in the three cell lines considered, K562, LAMA84 and KCL22, representative of the comparisons within the two treatment groups. (CTR: control, TKIs: Tyrosine Kinase Inhibitors) (Unpaired two-tailed t-test)

**Figure S7:** Expression of CD11b transcript (normalised for GAPDH) in the three cell lines considered, K562, LAMA84 and KCL22, representative of the comparisons within the two treatment groups. (CTR: control, TKIs: Tyrosine Kinase Inhibitors) (Unpaired two-tailed t-test)

**K562**

**CTR**

**LAMA84**

**KCL22**

**ASCI**

**B1**

**B2**

**B3**

**Figure S8:** Panel of images illustrating asciminib treatment in K562, LAMA84, and KCL22 cell lines obtained using the Invitrogen^TM^ EVOS^TM^ XL Core Imaging System with a 4X zoom. **Panel A1, A2, A3**: Control at 4X zoom in the three cell lines; **Panel B1, B2, B3**: asciminib at 4X zoom in the three cell lines. (CTR: control, ASCI: asciminib).
